# Expression analysis of inflammasomes in experimental models of inflammatory and fibrotic liver disease

**DOI:** 10.1186/1476-9255-9-49

**Published:** 2012-11-28

**Authors:** Sorina Georgiana Boaru, Erawan Borkham-Kamphorst, Lidia Tihaa, Ute Haas, Ralf Weiskirchen

**Affiliations:** 1 Institute of Clinical Chemistry and Pathobiochemistry, RWTH University Hospital Aachen, Pauwelsstr. 30, Aachen D-52074, Germany

**Keywords:** Hepatic inflammation, Inflammasome, Animal models, Hepatocytes, Hepatic stellate cells, Kupffer cells

## Abstract

During inflammation, the inflammasomes representing a group of multi-protein complexes trigger the biological maturation of pro-inflammatory cytokines such as interleukin-1β and interleukin-18 by proteolytic activation of caspase-1 from its inactive proforms. The individual genes encoding components of the inflammasome machinery are regulated at transcriptional and post-transcriptional levels. Once activated, they drive a wide variety of cellular responses that are necessary to mediate host defense against microbial pathogens and to guarantee tissue homeostasis. In the present work, we have studied the expression of the different inflammasomes in various primary hepatic cell subpopulations, in models of acute inflammation and during experimental liver fibrogenesis. We demonstrate that NLRP-1, NLRP-3 and AIM2 are prominently expressed in Kupffer cells and liver sinusoidal endothelial cells, moderately expressed in periportal myofibroblasts and hepatic stellate cells, and virtually absent in primary cultured hepatocytes. We found that the challenge with the lipopolysaccharides results in a time- and concentration-dependent expression of the NOD-like receptor family members NLRP-1, NLRP-3 and NLRC4/NALP4 in cultured hepatic stellate cells and a strong transcriptional activation of NLRP-3 in hepatocytes. Moreover, we detect a diverse regulatory network of the different inflammasomes in the chosen experimental models of acute and chronic liver insult suggesting that the various inflammasomes might contribute simultaneously to the outcome of inflammatory and fibrotic liver insult, irrespectively of the underlying inflammatory stimulus.

## Background

The inflammasomes are cytoplasmic multiprotein complexes that have recently been identified in immune cells as an important sensor of signals released by cellular injury and death. During inflammation they trigger the maturation of pro-inflammatory cytokines such as interleukin-1β (IL-1β) and engage innate immune defenses and function as a guardian of organ homeostasis [1-5]. Likewise, cellular stress, viral or bacterial infection, free cytoplasmic DNA, or any other kind of injury leads to activation of specialized receptors resulting in the formation of these high-molecular-mass inflammasome platforms [6-9]. The assembly of these molecular platforms is unique, triggered by a variety of endogenous and exogenous signals and follows a defined chronological sequence: after recognition of pathogen-derived molecules, the inflammasomes induce the autocatalytic generation of intracellular active caspase-1 (CASP-1) that in turn induces the proteolytic cleavage and biological activation of the IL-1β- and IL-18-precursors [6,7,10]. In principal, there are four individual inflammasomes (i.e. NLRP-1, NLRP-3, NLRC4/NALP4, and AIM2) that are each composed of a sensor molecule that is a member of the family of nucleotide-binding oligomerization domain (NOD)-like receptors (NLR) [11]. In its activated form, this sensor molecule has the ability to physically interact with CASP-1 or to recruit this protease *via* an intermediary adaptor molecule termed apoptosis-associated speck-like protein or caspase recruitment domain (CARD) of the adaptor protein ASC [4]. Once activated, a complex network of cellular reactions is triggered leading to local and systemic (e.g. acute-phase response) inflammatory reactions, recruitment of neutrophils and platelets as well as activation of the innate immune system [7]. Furthermore, the activation of the inflammasomes is linked to host defense against microbial pathogens, in many other multifaceted diseases such as metabolic syndrome and inflammatory bowel disease. In addition, inflammasomes are relevant in the regulation of diverse important aspects of inflammation and tissue repair such as pyroptosis representing a specialized form of cell death [5]. Based on these eminent functions, it is not surprising that mutations within this family of genes are associated with severe immune diseases and supposed to be involved in tumorigenesis [12]. Most studies highlighting the regulation and function of the different inflammasome branches are presently available from lung but it is now well documented that inflammasome activation is a general phenomenon found in all organs that is also proposed to be involved in insulin signaling, β-cell function and formation of atherosclerosis [5,13].

In regard to liver it has been recently demonstrated that the NLR family members NLRP6/NALP6 and NLRP-3 in conjunction with IL-18 negatively regulate progression of non-alcoholic fatty liver disease [14] and that the application of endotoxins including lipopolysaccharide (LPS) or fatty acids results in increased IL-1β production and strong activation of the NLRP-3 inflammasome [15,16]. Moreover, it was proposed that the induction and proteolytical activation of CASP-1 during activation of inflammasomes has hepatoprotective effect, in part through regulation of cell death pathways after major trauma [17]. In line, the silencing of NLRP-3 during liver ischemia-reperfusion by small hairpin RNAs confirmed that NLRP-3 signaling is involved in progression of liver injury and that its lack can protect the liver by reducing the concentration of IL-1β, IL-18, TNF-α, and IL-6 through downregulation of CASP-1 activation and NF-κB activity in mice [18]. At the cellular level, it was proposed that the activation of inflammasome components regulate a variety of endogenous functions in hepatic stellate cells (HSC) and are required for the development of liver fibrosis [19]. However, precise activities and involved signaling pathways of individual inflammasomes in liver cells are still enigmatic and the exact determination how the inflammasomes are activated in different diseases and experimental settings remains a demanding challenge.

Here we studied the inflammasome expression in various primary hepatic cell subpopulations and in experimental models of acute and chronic inflammation and ongoing hepatic fibrogenesis. We demonstrate that NLRP-1, NLRP-3 and AIM2 are prominently expressed in Kupffer cells (KC) and liver sinusoidal endothelial cells (LSEC), moderately expressed in periportal myofibroblasts (pMF) and HSC, and virtually absent in primary cultured hepatocytes. We further demonstrate that *in vitro* stimulation with LPS results in a time- and concentration-dependent activation of NLRP-1, NLRP-3 and NLRP4 in cultured HSC and a strong activation of NLRP-3 in hepatocytes. In summary, we found a dynamic transcriptional regulation of the diverse inflammasomes in experimental models of acute and chronic liver insult suggesting that the various inflammasomes might contribute alone or in conjunction with each other to the outcome of liver insult.

## Methods

### Cell culture

Primary HSC, KC and LSEC were isolated from male Sprague–Dawley rats by a standard Nycodenz density gradient centrifugation technique and cultured as described previously [20,21]. Fully transdifferentiated myofibroblasts (MFB) were obtained by subcultivation of HSC seven days after initial plating. Primary hepatocytes were isolated after the collagenase method established by Seglen [22] and plated and cultured for one day on collagen-coated dishes in HepatoZYME-SFM medium (Gibco, St Louis, MO, USA). pMF were prepared following established protocols [23], characterized by their microscopic appearance, their positivity for fibulin-2 and cultured essentially as described previously [24]. The rat cirrhotic fat storing cell line CFSC-2G [25,26] was cultured in Dulbecco’s modified Eagle medium (DMEM) containing 10% fetal calf serum (FCS) (Gibco), 4 mM L-Glutamine, 100 IU/ml penicillin, 100 μg/ml streptomycin, and 1 x non essential amino acids (all from Cambrex, Verviers, Belgium).

### LPS stimulation

For endotoxin stimulation, CFSC-2G were starved for 16 hr in DMEM containing 0.5% FCS (Gibco) and stimulation was done with indicated LPS concentrations in medium containing 0.2% FCS. When primary hepatocytes were stimulated with LPS, the cells were initially plated in HepatoZYME, then cultured in DMEM (10% FCS) overnight, followed by starvation for 8 h in DMEM (0.5% FCS), and stimulated with LPS as described above.

### RNA isolation and qRT-PCR

RNA from primary liver cells, rat cirrhotic fat storing cell line CFSC-2G or total liver tissue was isolated by the guanidine thiocyanate/CsCl method, followed by DNAse digestion using the Purelink RNA Mini kit system (Invitrogen, Life Technologies, Darmstadt, Germany). Total RNA was quantified and 2 μg samples reverse transcribed using Superscript II reverse transcriptase and random hexamer primers (both from Invitrogen). For the individual TaqMan PCR assays, the cDNA derived from 25 ng RNA was amplified in 25-μl volume using qPCR Core Kits (Eurogentec, Cologne, Germany) and primer combinations given in Table 1. The amplification of all respective target gene sequences were done as follows: melting at 95°C for 10 min and then 40 cycles at 95°C for 15 sec and 60°C for 1 min, respectively. Normalization was done either to theexpression of GAPDH or rS6 mRNAs.

**Table 1:** Primers used for quantitative TaqMan analysis

**Rat**		
**Gene of interest**	**GenBank no.**	** Primers**
**NLRP-1**	NM_001145755	For: 5’-gccctggagacaaagaatcc-3’
		Rev: 5’-agtgggcatcgtcatgtgt-3’
**NLRP-3**	NM_001191642	For: 5’-gctgtgtgaggcactccag-3’
		Rev: 5’-gaaacagcattgatgggtca-3’
**NLRC-4**	NM_001106707	For: 5’-ggccggaagtgaagctcta-3’
		Rev: 5’-cccctccagttgcttcag-3’
**AIM-2**	XM_222949	For: 5’-tggaaaccagagcaaaacaa-3’
		Rev: 5’-tgggctttgcagccttaata-3’
**IL-1β**	NM_031512	For: 5’-tgtgatgaaagacggcacac-3’
		Rev: 5’-cttcttctttgggtattgtttgg-3’
**IL-18**	NM_019165	For: 5’-cctgatatcgaccgaacagc-3’
		Rev: 5’-ccttccatccttcacagatagg-3’
**ASC**	NM_172322	For: 5’-gctcacaatgtctgtgcttagag-3’
		Rev: 5’-gcagtagccacagctccag-3’
**TNF-α**	NM_012675	For: 5’-gcccagaccctcacactc-3’
		Rev: 5’-ccactccagctgctcctct-3’
**rS6**	NM_017160	For: 5’-tgctcttggtgaagagtgga-3’
		Rev: 5’-caagaatgccccttactcaaa-3’
**Mouse**		
**Gene of interest**	**GenBank no.**	** Primers**
**NLRP-1a**	NM_001004142	For: 5’-attttgtggccctccaaga-3’
		Rev: 5’-ttgaaagtgggcaacatgg-3’
NLRP-1*	NM_001004142	For: 5’-tggcacatcctagggaaatc-3’
		Rev: 5’-tcctcacgtgacagcagaac-3’
**NLRP-3**	NM_145827	For: 5’-cccttggagacacaggactc-3’
		Rev: 5’-gaggctgcagttgtctaattcc-3’
**NLRC-4**	NM_001033367	For: 5’-tgatctccaagagatgaagttgg-3’
		Rev: 5’-gatcaaattgtgaagattctgtgc-3’
**AIM-2**	NM_001013779	For: 5’-tcaggaagttttcctttttctca-3’
		Rev: 5’-acagtcccaggatcagccta-3’
**IL-1β**	NM_008361	For: 5’-tgtaatgaaagacggcacacc-3’
		Rev: 5’-tcttctttgggtattgcttgg-3’
**IL-18**	NM_008360	For: 5’-caaaccttccaaatcacttcct-3’
		Rev: 5’-tccttgaagttgacgcaaga-3’
**ASC**	NM_023258	For: 5’-gagcagctgcaaacgactaa-3’
		Rev: 5’-gtccacaaagtgtcctgttctg-3’
**TNF-α**	NM_013693	For: 5’-tcttctcattcctgcttgtgg-3’
		Rev: 5’-ggtctgggccatagaactga-3’
**IL-6**	NM_031168	For: 5’-gctaccaaactggatataatcagga-3’
		Rev: 5’-ccaggtagctatggtactccagaa-3’
**IL-10**	NM_010548	For: 5’-ggctgaggcgctgtcatcg-3’
		Rev: 5’-tcattcatggccttgtagacacc-3’
**IFN-γ**	NM_008337	For: 5’-ggaggaactggcaaaaggatgg-3’
		Rev: 5’-tgttgctgatggcctgattgtc-3’
**CCL-2**	NM_011333	For: gtgttggctcagccagatgc-3’
		Rev: gacacctgctgctggtgatcc-3’
**GAPDH**	XM_001473623	For: 5’-actgccacccagaagactg-3’
		Rev: 5’-caccaccctgttgctgtag-3’
**rS6**	BC092050	For: 5’-cccatgaagcaaggtgttct-3’
		Rev: 5’-acaatgcatccacgaacaga-3’	

Note: * The primer combination for was taken from Ganz et al., WJG 2001;21:4772–8. It was taken to amplify NLRP-1 in hepatocytes. The NLRP-1a primer combinations were taken when NLRP-1 was amplified from total liver extracts.

### SDS-PAGE and immunoblotting

Whole-cell and liver protein extracts were prepared essentially as previously described [27]. Equal amounts of proteins (20 μg/lane) were resolved in NuPAGE^TM^ Bis-Tris gels (Invitrogen) and electroblotted on a Protran membrane (Schleicher & Schuell, Dassel, Germany). The sources and concentrations of antibodies used in Western blot analysis are given in Table 2.

**Table 2:** Antibodies used in this study

**Primary antibodies**				
**Protein/Antibody**	Clonality*	** Supplied by**	**Epitope, location**	**Dilution**
STAT1 (sc-346)	Poly	Santa Cruz Biotechnology, Santa Cruz, CA, USA	raised peptide mapping near the C-terminus of STAT1 p84/p91 of human origin	1 : 500
pSTAT1 (#9167)	Mono (r)	Cell Signaling, Technology, Danvers, MA, USA	phospho-STAT1 (Tyr701) (58D6), raised against synthetic phosphopeptide corresponding to residues surrounding Tyr701 of human STAT1	1 : 1,000
STAT3 (#4904)	Mono	Cell Signaling	raised a STAT3 fusion protein corresponding to the carboxy-terminal sequence of mouse Stat3 protein	1 : 1,000
pSTAT3 (#9134)	Poly	Cell Signaling	phospho-STAT3 (Ser727), raised against a synthetic phosphopeptide corresponding to residues surrounding Ser727 of mouse Stat3	1 : 1,000
NFκB (sc-8008)	Mono	Santa Cruz	NFκB, raised against amino acids 1–286 of NFκB p65 of human origin	1 : 1,000
pNFκB (#3033)	Mono	Cell Signaling	phospho NFκB; raised against a synthetic phosphopeptide corresponding to residues surrounding Ser536 of human NF-κB p65	1 : 1,000
JNK (#9252)	Poly	Cell Signaling	SAPK/JNK, raised against a GST/human JNK2 fusion protein	1 : 1,000
pJNK (#9251)	Poly	Cell Signaling	phospho-SAPK/JNK (Thr183/Tyr185), raised against a synthetic phosphopeptide corresponding to residues surrounding Thr183/Tyr185 of human SAPK/JNK	1 : 1,000
pJNK (#4668)	Mono (r)	Cell Signaling	phospho-SAPK/JNK (Thr183/Tyr185) (81E11), raised again a synthetic phosphopeptide (KLH-coupled) corresponding to residues surrounding Thr183/Tyr185 of human SAPK/JNK.	1 : 1,000
Casp-3 (#9664)	Mono (r)	Cell Signaling	cleaved Caspase-3 (Asp175) (5A1E), raised against a synthetic peptide corresponding to amino-terminal residues adjacent to Asp175 of human caspase-3	1 : 1,000
LCN2 (AF3508)	Poly	R&D Systems, Wiesbaden, Germany	recombinant rat Lipocalin-2/NGAL produced in mouse myeloma cell line NSO, polyclonal goat IgG	1 : 1,000
AIM-2 (14-6008-93)	Poly	eBioscience, San Diego, CA, USA	rabbit antibody that reacts with human, mouse, and rat AIM-2	1 : 1,000
NALP1 (4990)	Poly	Cell Signaling	human NALP1, raised against a synthetic peptide that corresponds to a region surrounding Gly1081 of human NALP1, crossreacts with human, mouse and rat NALP1	1 : 1,000
NALP3 (sc-66846)	Poly	Santa Cruz	Cryopyrin/NALP3 (H-66), rabbit antibody raised against amino acids 25–90 mapping near the N-terminus of human Cryopyrin	1 : 1,500
NALP4/NLRP4 (ab 47241)	Poly	Abcam, Cambridge, UK	raised against synthetic peptide corresponding to amino acids 139–157 of human NALP4	1 : 1,000
β-actin (#A5441)	Mono	Sigma-Aldrich, Taufkirchen, Germany	β-actin, synthetic peptide N-terminus (clone AC-15)	1 : 10,000
GAPDH (sc-32233)	Mono	Santa Cruz	GAPDH (clone 6C5)	1 : 1,000
**Secondary antibodies**				
sc-2004	NA	Santa Cruz	goat anti-rabbit IgG-HRP	1 : 5,000
sc-2056	NA	Santa Cruz	donkey anti-goat IgG-HRP	1 : 5,000
sc-2005	NA	Santa Cruz	goat anti-mouse IgG-HRP	1 : 5,000

* Abbreviations used for clonality are: Mono, monoclonal antibody produced in mouse; Mono (r), monoclonal antibody produced in rat; NA, not applicable; Poly, polyclonal antibody produced in rabbit.

### Experimental *in vivo* liver injury models

(i) Bile duct ligation (BDL) in rats: Male Sprague Dawley rats were subjected to bile duct ligation for 2, 7 or 14 days following a protocol previously described [28,29]. Sham operated rats that were sacrificed at the same time points served as controls. (ii) CCl_4_ application in rats: A total of 30 male Sprague Dawley rats at age six to eight weeks and weighing about 180–200 g were utilized for this study. The rats received intraperitoneal injections twice weekly of 1 ml/kg of CCl_4_ in an equal volume of mineral oil for up to 12 weeks, whereas mineral oil alone was used for 12 control animals following established protocols. Liver specimens were harvested and snap frozen and stored at −80°C for protein and RNA isolation. (iii) LPS and Concanavalin A (Con A) models in mice: Eight weeks old C57BL/6 wild type mice were subjected to a single intravenous injection of 20 mg/kg body weight Con A (Sigma) as previously described [30] or LPS (2.5 μg/g body weight). After 8 hrs (Con A) or after 2 or 6 hrs (LPS), mice were sacrificed and liver extracts prepared for qRT-PCR and Western blot analysis. All animal experiments performed were approved by the local review board according to prevailing guidelines for scientific animal experimentation.

### Immunohistochemistry of liver sections

Liver tissue sections were deparaffinized and rehydrated with xylene and decreasing graded ethanol, and antigen retrieval was engendered by heating the sections in 0.01 M sodium citrate buffer (pH 6) in a microwave for 20 min. Blocking of nonspecific binding sides and antigen detection was essentially done as described elsewhere [31]. The source of the NLRP-3, AIM2, and the NALP4/NLRP4 antibodies used for this analysis are given in Table 2. Stains with normal rabbit control serum served as negative controls. The specimens were briefly counterstained with hematoxylin and representative images made at a magnification of x200 using a Nikon Eclipse 80i microscope (Nikon, Düsseldorf, Gemany) equipped with the NIS Elements Vis software (version 3.22.01).

### Terminal transferase dUTP nick end-labelling assay (TUNEL)

For DNA fragmentation detection resulting from apoptotic signalling cascades, we used *In Situ* Cell Death Detection Kit Fluorescein (# 1684795, Roche Diagnostics, Mannheim, Germany) according to manufacturer’s instructions. The presence of nicks in the DNA of cultured cells was identified by terminal deoxynucleotidyl transferase (TdT), an enzyme that catalyzes the addition of labelling dUTPs. In brief, cells were seeded on glass slides and incubated with 200 ng/ml LPS for 30 min or 16 hrs. Thereafter, slides were rinsed with PBS, cells permeabilized in 0.1% Trition, 0.1% sodium citrate for 3 min on ice, and TUNEL reaction mixture added. As a negative control, the cells were only incubated with label solution and as a positive control, the cells were incubated after permeabilization with DNAse I Mix for 10 min. Cells were analyzed by fluorescence microscopy for direct fluorescein in a Leica DMLB microscope (Leica, Wetzlar, Germany) using the DISKUS software (version 4.50.1638) obtained from Carl H. Hilgers (Königswinter, Germany).

### LDH assay

The measurement of cell death was done in 96 well plate formats using the Cytotoxicity detection kit^+^ (LDH), version 5 (Roche) that allows detection of cell-mediated cytotoxicity and quantification of the cytotoxic potential of compounds. The assay was done essentially as outlined in the manufacturer’s instruction. Briefly, 100 μl of cell-free culture supernatants obtained from equal cell numbers (seeded at a density of 2.5 x 10^5^ cells/well in 6 well-plates) that were incubated with different concentration of LPS were incubated with 100 μl of the reaction mixture from the test system kit. After incubation for 25 min at room temperature, 50 μl stop solution was added, the plates were shacked for 10 min and the formation of formazan was measured in a Wallac 1420 Victor Multilabel Counter (Wallac Oy, Turku, Finland) equipped with a 490 nm filter and the WIACALC (version 1) software. For standardization of LDH activity, purified LDH from hog muscle (Roche, #101107085001, Lot no. 12169925) with a specific activity of 597 U/mg was included in this analysis.

### Measurement of influx of neutrophils, monocytes and other immune cells in livers of mice subjected to BDL or CCl_4_ treatment *via* flow cytometry

To determine the influx of leukocytes into the livers of animals that received BDL surgery or were injected with CCl_**4**_, liver leukocyte isolates were prepared and analyzed by Fluorescence activated cell sorting (FACS). Individual leukocyte subsets were identified by their positivity for CD45 (all leukocytes), CD11b (monocytes and granulocytes), CD11b F4/80 (macrophages), and Ly-6G (neutrophils). A FACS Canto-II (BD Biosciences, Heidelberg, Germany) was used for flow cytometric analysis. The acquired data sets were analyzed by FlowJo software (TreeStar, Ashland, OR) and individual cell subsets depicted in percentage of all cells measured. The analysis shown was done from livers of animals taken 5 days after BDL surgery and 48 h after single CCl_4_ injection.

### Statistical analysis

Statistical analyses was performed using the t-test for comparison of groups and the Kruskal-Wallis test for nonparametric multiple comparison with a statistical software program (STATGRAPHICS Plus, version 5.1). When appreciable, results are depicted with their median values. Probability values of less than 0.05 or 0.01 were considered as statistically significant. To establish the differences within individual groups, we used the Mann–Whitney test. Individual p-values for each experiment are given in Additional file 1.

## Results

### Establishment of quantitative real time PCR (qRT-PCR) assays for analysis of inflammasomme expression in mouse and rat

There are several key regulatory genes involved in the initiation and regulation of inflammatory pathways. To allow relative mRNA quantification of the different components in mice and rats, we have established methodologies for measurement of NLRP-3, NRLC4, AIM2, IL-1β, IL-18, ASC, TNF-α, and rS6 in rat (Additional file 2: Figure S1) and NLRP-1b, NLRP-1c, NLRP-3, NLRC4/NALP4, AIM2, IL-1β, IL-18, ASC, TNF-α, IL-6, IL-10, IFN-γ, and GAPDH in mice (Additional file 3: Figure S2). In addition, we have established a protocol for measuring CCL2/MCP-1 mRNA expression in both species (not shown). These assays are designed for quantitative analysis or mRNA expression using the TaqMan platform. The different primer combinations (Table 1) that are used for amplification are designed in a fashion that allows amplification of respective target gene sequences under same cycling conditions (melting temperature: 95°C and amplification/extension temperature 60°C) permitting a general, easy applicable testing system of respective components.

### Expression of NLR family members in primary hepatic cell subpopulations

**Figure 1:**
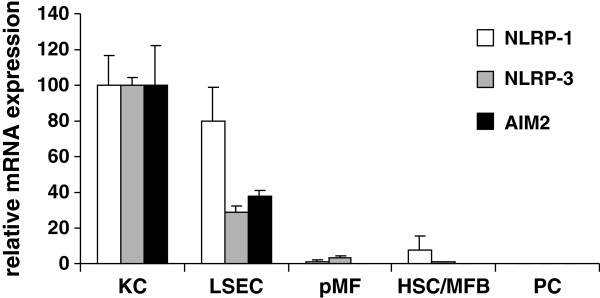
**Expression of NLRP-1, NLRP-3 and AIM2 in primary hepatic cells. **Total RNA from Kupffer cells (KC) cultured for 2 days, freshly isolated sinusoidal endothelial cells (LSEC), portal fibroblasts (pF) in primary culture, hepatic stellate cells (HSC/MFB) cultured for 4 days and hepatocytes (PC) cultured for 2 days were isolated and subjected to quantitative real time PCR for NLRP-1, NLRP-3, and AIM2. In this analysis, the expression of the different genes was set to 100% in KC. The individual primers used for quantitative analysis are depicted in Table 1. For detailed statistical analysis of this set of experiments see Additional file 1.

To get a first hint of inflammasome expression in the different hepatic cell subpopulations, we isolated KC, LSEC, pMF, HSC/MFB, and hepatocytes and quantified the relative expression of NLRP-1, NLRP-3, and the interferon-inducible HIN200 family member AIM2 in these cell entities. This analysis revealed that all three genes are highest expressed in KC and LSEC, while the expression was only weak in cultured pMF and HSC/MFB (Figure 1).

### Transcriptional regulation of inflammasome related genes in CFSC-2G after LPS challenge

**Figure 2:**
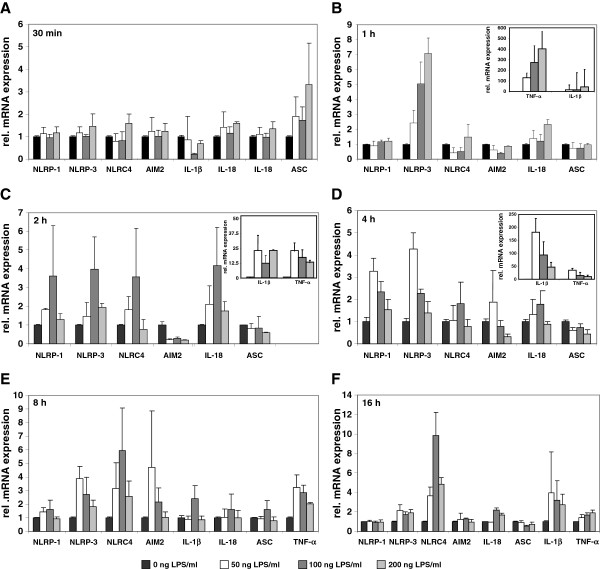
**Expression of inflammasome components in rat cirrhotic fat storing cell line CFSC-2G subjected to LPS stimulation. **CFSC-2G cells were treated one day after plating with 0, 50, 100 or 200 ng/ml LPS. After 30 min (**A**), 1 h (**B**), 2 hrs (**C**), 4 hrs (**D**), 8 hrs (**E**), 16 hrs (**F**), RNA from respective cells was extracted and, the expression of NLRP-1, NLRP-3. NLRC-4, AIM2, IL-1β, IL-18, ASC, and TNF-α analysed by qRT-PCR. In this analysis, the expression of each gene without LPS stimulation was set to 1. Please note, the high expression of TNF-α at early (inlets in **B** and **C**) and IL-1β (inlet in **D**) and medial time points after LPS treatment. For detailed statistical analysis of this set of experiments see Additional file 1.

It is well established that the endotoxin LPS plays an important modulator role in acute liver injury as well as chronic liver diseases. In addition, it was recently demonstrated that components of the inflammasome are expressed in immortalized and primary HSC and that the lack of ASC and NLRP-3 interfere with the process of ongoing fibrogenesis after CCl_4_ or thioacetamide (TAA) challenge that become apparent in reduced collagen deposition and HSC activation in mice lacking ASC or NLRP-3 [19]. To analyze the expression of relevant inflammasomes after inflammatory stimuli in more detail, we challenged cultured CFSC-2G cells with different concentrations of LPS (50, 100, and 200 ng/ml) and measured the mRNA expression of NLRP-1, NLRP-3, NLRC-4, AIM2, IL-1β, IL-18, ASC, and TNF-α after 30 min, 1 h, 2 hrs, 4 hrs, 8 hrs, and 16 hrs (Figure 2). As expected, the analysis shows a rapid increase in TNF-α expression that was highest after 1 h and strongly dependent on the concentration of LPS. In addition, we noticed a simultaneous upregulation of IL-1β expression. The expression of all four inflammasomes was not altered after 30 min of LPS challenge (Figure 2A).

However, one hour after LPS stimulation, the expression of NLRP-3 was dose-dependently upregulated, while all other genes were not affected at that time point. Interestingly, two hours after LPS stimulation the expression of NLRP-1, NLRP-3, NLRC4/NALP4 were significant upregulated to a similar degree, while the expression of AIM2 was lowered in cells that received LPS (Figure 2C). The highest expression of these components at that time point was observed in cells that were triggered with 100 ng LPS/ml culture medium, while even higher concentrations did not significantly affect the expression of these genes. At later time points, we only noticed a sustained higher expression of NLRC4/NALP4 and IL-1β (Figure 2F) suggesting that the different inflammasomes and genes that trigger inflammasome activity have a well defined individual expression kinetic after stimulation with LPS.

To rule out that LPS induced cytotoxic effects in CFSC-2G cells under the chosen experimental conditions that might interfere with the expression of the individual inflammasome genes, we further measured cell death and cell lysis by use of TUNEL assay (Additional file 4: Figure S3) and a cytotoxicity detection assay that is based on measurement of lactate-dehydrogenase (LDH) activity released from the cytosol of damaged cells (Additional file 5: Figure S4). Both assays revealed that the incubation of the cells with LPS does not result in plasma-membrane damage or activation of endonucleases that would be indicative for cells that undergo apoptosis.

### mRNA expression of inflammasomes during experimental liver insult induced by ligation of the common bile duct

**Figure 3:**
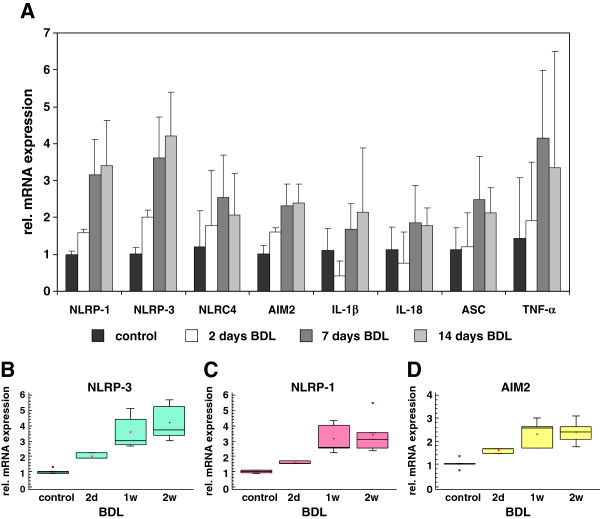
**Expression of inflammasome components in rat livers after bile duct ligation (BDL).** (**A**) Sprague Dawley rats were subjected to BDL and sacrificed 2 (3 animals), 7 (5 animals) or 14 (5 animals) days after surgery. The livers of 5 untreated rats were taken as controls. RNA was isolated and the expression of NLRP-1, NLRP-3. NLRC4/NALP4, AIM2, IL-1β, IL-18, ASC, and TNF-α analyzed by qRT-PCR. In this analysis, the expression of the tested genes in livers of sham operated rats (control) was set to 1. (**B**, **C**, **D**) Kruskal-Wallis testing of NLRP-3 (**B**), NLRP-1 (**C**), and AIM2 (**D**) expression during ongoing fibrogenesis induced by the BDL surgery. For detailed statistical analysis of this set of experiments see Additional file 1.

To analyze the expression of inflammasome genes during the process of acute and chronic liver insult, we first monitored expression of relevant genes in rats that underwent bile duct ligation (BDL). This model represents a well established experimental model of cholestatic liver disease. We performed qRT-PCR using RNA that was isolated from rats after 2, 7 or 14 after BDL surgery and compared the expression of the different genes to those observed in livers of sham-operated control rats. This analysis revealed that the expression of all tested genes (NLRP-1, NLRP-3, NLRC4/NALP4, AIM2, IL-1β, IL-18, ASC, and TNF-α) was increased in inflamed liver at all time points during ongoing insult (i.e. fibrogenesis) suggesting that the expression of these genes is not only necessary for disease initiation but also for progression (Figure 3A). A detailed statistical analysis using the Kruskal-Wallis test for nonparametric multiple comparison revealed that the observed increased expression of NLRP-3, NLRP-1 and AIM2 reached statistical significant values (Figures 3B, 3C, 3D).

### mRNA expression of inflammasomes during experimental liver insult induced by single or repeated application of CCl_4_

**Figure 4:**
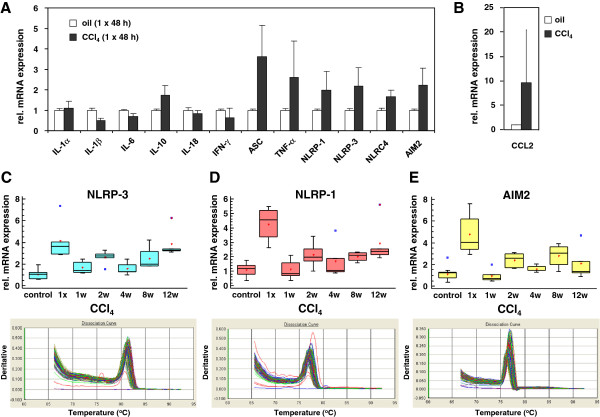
**Expression of inflammasome components in rat livers after application of CCl**_**4**_**. **(**A**) Sprague Dawley rats received one single application of CCl_4 _or oil. 48 hrs later RNA was prepared and analyzed for expression of IL-1α, IL-1β, IL-6, IL-10, IL-18, IFN-γ, ASC, TNF-α, NLRP-1, NLRP-3, NLRC-4 and AIM2 by qRT-PCR. Details on primer combinations are given in Table 1. (**B**) The same set of RNAs from (**A**) was analyzed for expression of CCL-2. (**C**) The livers of Sprague Dawley rats (3 animals/group) that received one single (1x) or twice weekly intraperitoneal injections of 1 ml/kg BW of CCl_4 _in an equal volume of mineral oil for 1, 2, 4, 8, or 12 weeks were sampled, RNA extracted, and the expression of NLRP-3 (**C**), NLRP-1 (**D**), and AIM2 (**E**) analyzed by qRT-PCR. Representative melting curves for the expression studies of the three genes investigated are depicted in the lower panels. For detailed information about primer combinations and statistical analysis of this set of experiments see Table 1 and Additional file 1.

We next tested the expression of various genes of the inflammasome machinery in mice 48 hrs after CCl_4_ injection. Compared to mice that received oil, the single administration of CCl_4_ resulted in significant increased expression of ASC, TNF-α, NLRP-1, NLRP-3, NLRC4/NALP4 and AIM2 (Figure 4A, see Additional file 1 for statistics) demonstrating that the single application of CCl_4_ is already suitable to induce a strong inflammatory reaction in liver. Under these conditions, we also observed an elevated expression of CCL2/MCP-1 (Figure 4B) that is known to be necessary for recruitment of monocytes, memory T cells, and dendritic cells to sites of tissue injury, infection, and inflammation [32,33]. When we analyzed expression of NLRP-3 and NLRP-1 and AIM2 in livers of animals that were subjected to prolonged treatment with CCl_4_ for 1, 2, 4, 8 and 12 weeks, we noticed that the initial high expression of all three genes were somewhat blunted at later time points and showed a somewhat undulated expression kinetics (Figure 4C).

### Protein expression of inflammasomes during experimental liver insult

**Figure 5:**
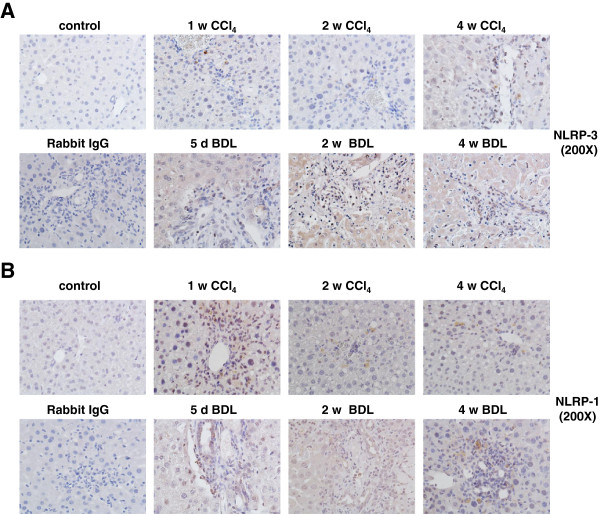
**Hepatic expression of NLRP-3 and NLRP-1 in rats after administration of CCl**_**4 **_**or BDL surgery. **Liver specimen from animals that received CCl_4 _or underwent BDL for indicated time points were stained with antibodies specific for NLRP-3 (**A**) or NLRP-1 (**B**). Section from control animals and a stain with an unspecific rabbit IgG served as controls in this analysis.

**Figure 6:**
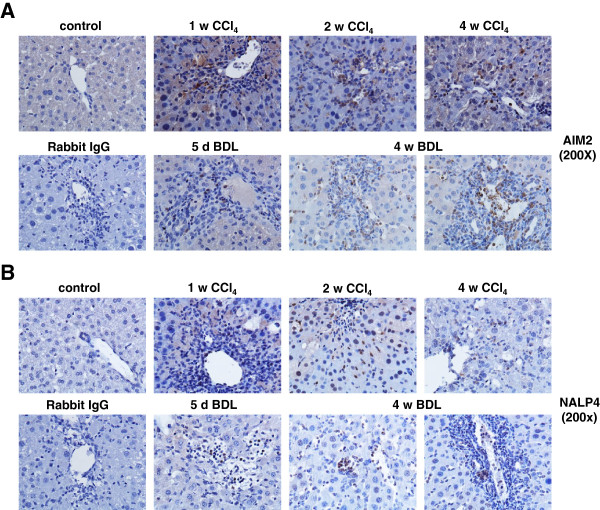
**Hepatic expression of AIM2 and NALP4/NLRC4 in rats after administration of CCl**_**4 **_**or BDL surgery. **Liver specimen from animals that received CCl_4 _or underwent BDL for indicated time points were stained with antibodies specific for AIM2 (**A**) or NALP4/NLRC4 (**B**). Section from control animals and a stain with an unspecific rabbit IgG served as controls in this analysis.

**Figure 7:**
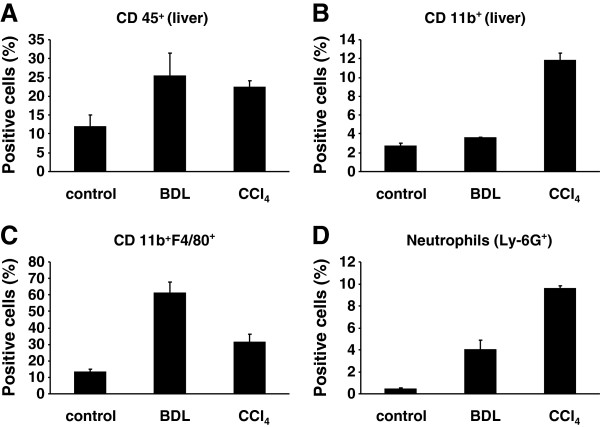
**Influx of neutrophils, monocytes and other immune cells in livers of mice subjected to either BDL or CCl**_**4 **_**treatment. **(**A**) Total number of leukocytes in liver cell extracts was identified by their positivity for CD45. (**B**) The numbers of monocytes and granulocytes were measured by their positivity for CD11b. **(C) **Macrophages were identified by their positivity for CD11b and F4/80. (**D**) The total number of neutrophils was measured by their positivity for Ly-6G. The analysis was done from livers of animals taken 5 days after BDL surgery and 48 h after single injection of CCl_4_.

To test if the observed mRNA expression kinetics of the different inflammasomes after administration of CCl_4_ or BDL surgery was also reflected at the protein level, we performed immunohistochemistry for NALP3, NALP1, AIM2, and NLRC4/NALP4 (Figures 5 and 6). In liver sections that were prepared from animals that received either BDL for 5 days, 2 weeks, and 4 weeks or subjected to CCl_4_ for 1 week, 2 weeks or 4 weeks, we found that hepatocytes stained weakly positive for NLRP-3 (Figure 5 A). In addition, another cell population that was most likely KC stained strongly positive for NLRP-3. In contrast, NLRP-3 was virtually absent in liver sections taken from untreated controls. Similar results were found for NALP1 (Figure 5B), AIM2 (Figure 6A), and NLRC4/NALP4 (Figure 6B) suggesting that there is (i) either an intensive influx of cells into liver with capacity to express these proteins, (ii) an increased expression of inflammasome proteins in liver residential cells (e.g. KC), or (iii) both cellular influx and enhanced expression by hepatic cell subpopulations. By FACS-based analysis of hepatic leukocyte isolates, we were able to demonstrate that in both models (i.e. BDL, CCl_4_) significant quantities of leukocytes infiltrated the liver (Figure 7A). While the quantities of monocytes/granulocytes (Figure 7B) were higher in the CCl_4_ model, livers of BDL animals showed a marked increase of macrophages (Figure 7C), while the number of neutrophils was higher in both models compared to untreated control livers (Figure 7D).

### Expression of inflammasomes and signaling cascades activated during acute inflammatory stimuli

**Figure 8:**
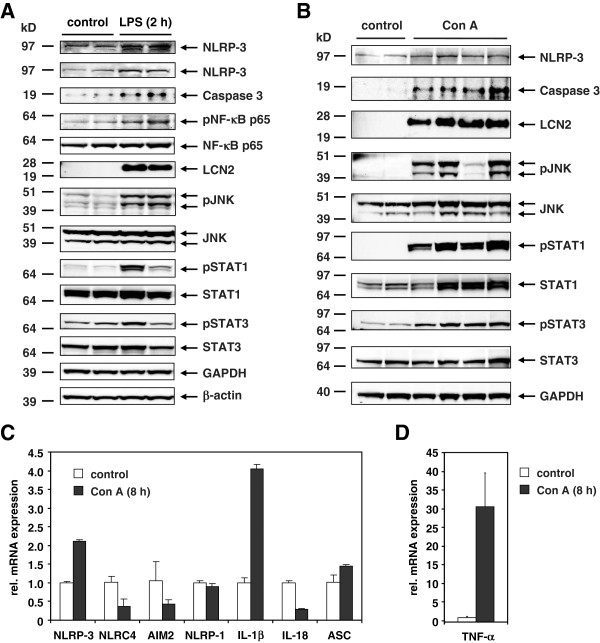
**Expression of inflammasomes in mice after LPS and Con A injection. **(**A**) Liver extracts were prepared from control mouse (4 animals) or animals that received intraperitoneal injection of LPS (2.5 μg/g body weight) for 2 (2 animals) or 6 hrs (5 animals). Equal protein amounts (100 μg) were then subjected to SDS-PAGE and analyzed in Western blot for expression of NLRP-3, Caspase 3, pNF-κB p65, total NF-κB p65, Lipocalin-2 (LCN2), pJNK, total JNK, pSTAT1, total STAT1, pSTAT3, total STAT3, GAPDH, and β-actin. A representative blot is shown for two controls and two animals that received LPS for 2 hrs. (**B**) Liver extracts were prepared from control mouse or animals that received intraperitoneal injection of Con A (20 μg/g body weight) for 8 hrs. Equal protein amounts were then subjected to SDS-PAGE and analyzed for expression of NLRP-3, Lipocalin-2 (LCN2), pJNK, total JNK, pSTAT1, total STAT1, pSTAT3, total STAT3, and GAPDH. The antibodies used in this study are given in Table 2. (**C**) RNA was isolated from livers of mice that were treated with Con A for 8 hrs or from 2 controls and 4 Con A-treated mice and analyzed for expression of NLRP-3, NLRC-4, AIM2, NLRP-1, IL-1β, IL-18, and ASC. (**D**) The same set of RNAs was tested for TNF-α expression. For detailed statistical analysis of this set of experiments see Additional file 1.

All these previous findings described above demonstrate that various stimuli that induce liver damage are in principle suitable to transcriptional activate the different inflammasome branches simultaneously suggesting that the subsequent molecular pathways regulated by these pathways are highly complex especially at later time points when secondary effects come along. Therefore, we decided to exemplary analyze NLRP-3 expression during the acute phase response. This reaction occurs soon after setting of inflammatory stimuli and represents a complex series of reactions resulting in the production of inflammation-associated cytokines and diverse acute phase proteins. For induction we either used the application of LPS or Concanavalin A (Con A) for 6 hrs (LPS) or 8 hrs (Con A). When we analyzed the expression of NLRP-3 in liver protein extracts from animals that received LPS, we found that the endotoxin induced expression of NLRP-3 at the protein level (Figure 8A). The LPS injection further resulted in a significant induction of the acute phase protein Lipocalin 2 (LCN2) and correlated well with the phosphorylation of the Signal Transducers and Activators of Transcription-1 (STAT1) that is involved in mediating the acute phase response *via* transcriptional activation of cytokine inducible genes. In line with this activation, we found that the LPS injection resulted in a significant phosphorylation of the JNK-MAPK and NF-κB/p65 and an increased expression of caspase-3. Significant phosphorylation of STAT-3 was already observed at 2 hrs after LPS injection (not shown) confirming well established findings that both STAT-1 and STAT-3 have critical roles in the control of systemic inflammation and acute phase response [34,35]. The induction of the acute phase response was further confirmed by quantitative analysis of various cytokine mRNA expression measuring after 2 and 6 hrs after application of LPS (Additional file 6: Figure S5A) and increased expression of CCL2/MCP-1 (Additional file 6: Figure S5B).

In the other acute model (i.e. Con A), we again found a strong upregulation of NLRP-3 that correlated well with increased expression of LCN2, caspase-3 and elevated phosphorylation of JNK and STAT-1 (Figure 8B). In this model, the increase of NLRP-3 expression seems to be the result of an increased transcriptional activation of the *Nlrp-3* gene (Figure 8C) and correlated well with the expression of IL-1β and TNF-α that are indicative for the acute phase response that is triggered by the LPS stimulus (Figure 8D). Interestingly, the expression of NLRC4/NALP4 and AIM2 was reduced after application of Con A, while NLRP-1 gene expression was unaffected.

### Induction of inflammasome expression in primary cultured hepatocytes and KC

**Figure 9:**
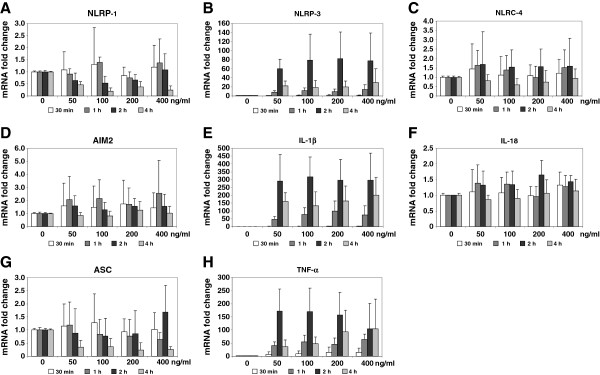
**Expression of inflammasomes in primary murine hepatocytes after stimulation with LPS.** Primary hepatocytes in culture were stimulated one day after isolation with 50, 100, 200 and 400 ng/ml LPS or left untreated. After indicated times (30 min, 1 h, 2 hrs, 4 hrs) RNA was isolated from respective cells and tested for expression of NLRP-1 (**A**), NLRP-3 (**B**), NLRC-4 (**C**), AIM2 (**D**), IL-1β (**E**), IL-18 (**F**), ASC (**G**), and TNF-α (**H**) by qRT-PCR. For detailed statistical analysis of this set of experiments see Additional file 1.

In the initial analysis, we found that the expression of inflammasomes in primary cultured hepatocytes is rather low or even not detectable (cf. Figure 1). However, we noticed *in vivo* that hepatocytes stained positive for NLRP-3 when animals were subjected to BDL (cf. Figure 3E) suggesting that hepatocytes should in principal be also able to respond to inflammatory stimuli with expression of NLRP-3 and other inflammasome-associated proteins. To test this in more detail, we serum-starved primary isolated murine hepatocytes to avoid influences of serum factors and stimulated them with different concentration of LPS for various time intervals and measured the expression of respective genes (Figure 9).

While the expression of NLRP-1 (Figure 9A), NLRC4/NALP4 (Figure 9C), AIM2 (Figure 9D), and ASC (Figure 9G) was nearly unaffected 30 min after stimulation with 50-400 ng LPS/ml medium, the expression of all four genes were more or less suppressed at later time points. In contrast, the LPS challenge resulted in a drastic upregulation of NLRP-3 expression (up to 100 fold two hours after stimulation) at all concentrations tested (Figure 9B). This expression pattern correlated well with the expression of IL-1β (Figure 9E) and TNF-α (Figure 9H). The analysis further revealed that the mRNA quantity of caspase-1 that represents the major protease necessary to activate the inflammasome machinery is also highest at 2 hrs, while the expression of IL-18 in hepatocytes is unaffected after LPS challenge.

In this scenario it is most likely that liver residential KC are the major source of IL-1β and TNF-α because it is generally assumed and confirmed by our analysis (Additional file 7: Figure S6) that primary isolated KC have a high capacity to express these pro-inflammatory cytokines after LPS stimulation.

## Discussion

There is no doubt that the understanding of inflammasome regulation and function will potentially offer great opportunities to interfere with the process of inflammation, fibrogenesis and tumorigenesis. Irrespectively of the inflammatory stimuli analyzed, the activation of the inflammasome machinery is a well orchestrated process in which pattern recognitions receptors recognize distinct danger signals and in turn activate signaling pathways that subsequently initiate the inflammatory response resulting in activation of different pathways such as NF-κB and MAPK and culminating in transcriptional activation of a large number of different inflammation-associated genes. It is superfluous to mention and confirmed in our study that this regulatory network is highly complex and that the individual inflammasome protein complexes might be simultaneously expressed and activated in the same cell type at the same time in the inflamed tissue. Recent studies have characterized and classified distinct molecular agents and pathways for several sensor proteins and have identified a multitude of inflammatory ligands of both endogenous and exogenous origin that drive inflammasome activities in healthy and diseased organs [5]. However, there is only limited knowledge of inflammasome regulation and function in healthy liver and various liver diseases.

To allow quantification of mRNAs of genes that are directly linked to the activity of the inflammasomes, we have established qRT-PCR assays for mRNA quantification of NLRP-3, NLRP-1, NLRC4/NALP4, AIM2, IL-1β, IL-18, ASC, TNF-α, IL-6, IL-10 in mice and rat (Additional file 2: Figure S1, Additional file 3: Figure S2).

We have shown that the expression of NLRP-1, NLRP-3 and AIM2 in cultured primary cell population is mainly restricted to KC, LSEC and pMF, while the expression in HSC is only low and virtually absent in primary cultured hepatocytes (Figure 1). However, the challenge with LPS demonstrates that the expression of respective genes involved in formation of inflammasomes can be induced to high levels in HSC/MFB (Figure 2) and hepatocytes (Figure 6) suggesting that all liver cell types tested are in principal able to mediate inflammasome activities. Interestingly, NLRP-3, NLRP-1 and NLRC4/NALP4 are inducible in CFSC-2G in response to LPS stimulation (Figure 2) demonstrating that the different inflammasome branches can be simultaneously activated at the same time in this cell entity. However, during activation there seems to be a clear sequential order because NLRP-3 expression was found to be highest already one hour after LPS challenge, while the elevated expression of NLRP-1 and NLRC4/NALP4 followed one hour later.

During experimental liver insult induced by ligature of the common bile duct (BDL), the expression of all four core inflammasomes (i.e. NLRP-1, NLRP-3, NLRC4/NALP4, and AIM2) were simultaneously activated at the mRNA (Figures 3) and protein level (Figures 5 and 6). Also the single or repeatedly application of CCl_4_ resulted in a simultaneous increase of all four inflammasomes in liver (Figure 4, Figures 5 and 6) suggesting that inflammatory stimuli induce a highly complex network of biological responses in residential and infiltrating cells in which all inflammatory branches are integrated.

When mice were injected with LPS, the expression of NLRP-3 was induced at both mRNA and protein levels (Figure 5A) confirming previous reports [16]. LPS is a prototypical ligand for the Toll like receptor 4 (TLR4) that upon activation induces the production of pro-inflammatory cytokines through activation of the NF-κB pathway [36,37]. In addition, we observed a strong induction/activation (i. e. phosphorylation) of other genes in that disease model which are involved in inflammatory reactions and recovery from endotoxic shock including LCN2, caspase-3, NF-κB, JNK, pSTAT1, and STAT3 [35,38]. A comparable activation pattern of all these genes was observed when normal liver was challenged with Con A (Figure 5B, 5C). Since Con A injection leads to immune-mediated liver injury and release of several cytokines (e.g. TNF-α, IFN-γ) triggering liver damage [39], these findings suggest that irrespectively of the stimuli triggering the inflammatory response, the subsequent changes within the liver end up in similar molecular alterations and correlate with the activation of the NLRP-3 inflammasome.

Presently, we do not know if the induction/activation of the diverse target genes and pathways occur in an orchestrated way with NLRP-3 or if the activation of these genes is mandatory to stimulate NLRP-3 expression. However, based on our experimentation, it is reasonable to speculate that the expression of inflammasome components is directly linked to the activation of NF-κB. It is known that under the condition of BDL, rats have an overall constitutive activation of NF-κB in the liver [40]. Also carbon tetrachloride exposure in mice leads to activation of NF-κB [41]. Likewise, the application of the lectin Con A and endotoxin LPS induces activation and nuclear translocation of NF-κB [36,42]. Therefore, it will be interesting to test if the administration of inhibitors of canonical or non-canonical NF-κB signaling such as the thiol-reactive quinol and putative thioredoxin inhibitor PMX464 or the lack of factors necessary to activate/phosphorylate NF-κB is suitable to interfere or blunt expression of inflammasome genes.

The finding that purified hepatocytes alone do not express inflammasome components (Figure 1) but induce their transcription after appropriate challenge with inflammatory stimuli such as LPS (Figure 6) demonstrate that the presence of immune cells *per se* is not necessary to mediate respective responses *in vitro*. However, it will be essential to analyze *in vivo* in more detail if the initiation of inflammasome activity in inflamed liver tissue is mainly triggered by influx of neutrophils, monocytes and other immune cells, is a capacity of liver residential cells or is the outcome of both processes. Based on our findings, we suggest that infiltrating cells as well as liver residential cells have capacity to induce inflammasome expression after appropriate trigger within the liver. Most likely, all primary hepatic cell entities are capable to induce inflammasome expression and act in conjunction with infiltrating cells that may vary in the different experimental settings.

The fact that the immortalized cirrhotic fat storing cell line CFSC-2G induces the inflammasome machinery after challenge with LPS (Figure 2) further demonstrates that immortalization is not sufficient to blunt inflammasome activity.

Definitely, we are still at the beginning in understanding the regulation of inflammasomes in different disease models and far away to understand the functions of the individual components. The fact that the expression of the different inflammasome branches in liver become simultaneously activated during hepatic inflammation and their linkage to the activation of general molecular transcriptions factors (e.g. NF-κB) further strengthens the notion that there are several master key switches of inflammasome activity. It will be interesting and challenging to unravel these interactions and to identify specific regulatory control points that might be suitable for pharmacological intervention.

## Conclusions

In regard to liver NLRP-1, NLRP-3 and AIM2 are most prominently expressed in Kupffer cells and liver sinusoidal endothelial cells, moderately expressed in periportal myofibroblasts and hepatic stellate cells, and virtually absent in primary cultured hepatocytes. *In vitro*, the challenge of cultured hepatic stellate cells with lipopolysaccharides results in a time- and concentration-dependent upregulation of NLRP-1, NLRP-3 and NLRC4/NALP4, while hepatocytes respond mainly with a strong transcriptional activation of NLRP-3. Our data using different experimental animals of inflammatory liver insult further indicates that the various inflammasome components contribute simultaneously to the outcome of inflammatory liver disease, irrespectively of the underlying inflammatory stimuli. The fact that the stimulation of all inflammasome branches in the different disease models leads to simultaneous activation of general molecular transcriptions factors (e.g. NF-κB) further strengthens the notion that there are several master key switches regulating inflammasome activity.

## Abbreviations

BDL: Bile duct ligation; CASP-1: Caspase-1; Con A: Concanavalin A; FACS: Flurorescence-activated cell sorting; HSC: Hepatic stellate cell(s); KC: Kupffer cell(s); LPS: Lipopolysaccharides; LSEC: Liver sinusoidal endothelial cell(s); MFB: Myofibroblasts; NLR: Nucleotide-binding oligomerization domain (NOD)-like receptors; PC: Parenchymal cell(s), i. e. hepatocyte(s); pMF: periportal myofibroblast(s).

## Competing interests

The authors declare that they have no competing interest.

## Authors’ contributions

SGB, EBK, LT, and UH performed the experiments and designed figures; RW designed the study and drafted the manuscript. All authors read and approved the final manuscript.

## Supplementary Material

Additional file 1 **Statistics to Figure 2. **Expression of inflammasome components in rat cirrhotic fat storing cell line CFSC-2G subjected to LPS stimulation. Statistics to Figure 3: Expression of inflammasome components in rat livers after bile duct ligation (BDL). Statistics to Figure 4: Expression of inflammasome components in rat livers after application of CCl_4 _. Statistics to Figure 5: Expression of inflammasomes in mice after Con A injection. Statistics to Figure 6: Expression of inflammasomes in primary murine hepatocytes after stimulation with LPS. Statistics to Suppl. Figure 3: Induction of acute phase response in mice after LPS injection. Click here for file

Additional file 2: Figure S1 Establishment of TaqMan tests for expression analysis of individual inflammasome genes in rats. TaqMan assays for rat NLRP-3 **(A)**, NLRC-4 **(B)**, AIM2 **(C)**, IL-1β **(D)**, IL-18 **(E)**, ASC **(F)**, TNF-α **(G)**, and rS6 **(H) **were established. Representative melting curves for each gene are depicted. Amplification of respective target gene sequences were performed under the same cycling conditions using a melting temperature of 95°C and amplification/extension temperatures of 60°C, respectively. The individual primer combinations used in each test are depicted in Table 1. Click here for file

Additional file 3: Figure S2 Establishment of TaqMan tests for expression analysis of individual inflammasome genes in mouse. TaqMan assays for murine NLRP-1b **(A)**, NLRP-1c **(B)**, NLRP-3 **(C)**, NLRC-4 **(D)**, AIM2 **(E)**, IL-1β **(F)**, IL-18 **(G)**, ASC **(H)**, TNF-α **(I)**, IL-6 **(J)**, IL-10 **(K),** IFN-γ **(L) **and GAPDH **(M) **were established. Amplification of the different gene sequences were essentially performed under the same cycling conditions each using a melting temperature of 95°C and amplification/extension temperatures of 60°C, respectively. Representative melting curves for each gene are depicted. The individual primer combinations used in each test are depicted in Table 1. Click here for file

Additional file 4: Figure S3 Analysis of DNA fragmentation after treatment with LPS. **(A)** TUNEL assay in CFSC-2G cells that were stimulated in short (30 min) and long term (16 h) with 200 ng/ml LPS kept their cellular integrity. The nuclei are counterstained with DAPI. Click here for file

Additional file 5: Figure S4 Analysis of cell cytotoxicity after treatment with LPS. **(A) **The standard curve for measurement of LDH activity was established with a preparation of hog LDH. **(B) **The chosen kit system allows to measure LDH activity in a wide range from 0.001 (Background), to 5,083 (high control). **(C) **Measurement of LDH in cellular supernatants of CFSC-2G cells that were incubated with indicated concentrations of LPS for indicated time intervals reveal only low cellular toxicity of LPS. Click here for file

Additional file 6: Figure S5 Induction of acute phase response in mice after LPS injection. **(A) **Mice were subjected to LPS stimulation and livers samples were taken after 2 and 6 hours. The expression of pro-inflammatory, anti-inflammatory cytokines as well as indicated inflammasome components was analyzed by qRT-PCR. **(B) **Expression analysis of the monocyte chemotactic protein (MCP-1, CCL2) in livers after LPS injection. For detailed statistical analysis of this set of experiments see Additional file 1. Click here for file

Additional file 7: Figure S6 Expression analysis of Kupffer cells after stimulation with LPS. Primary KC were stimulated with indicated concentration of LPS for 2 hrs and the expression of **(A) **NLRP-1, NLRC4/NALP4, AIM2, IL-18, ASC and **(B) **NLRP-3, IL-1β and TNF-α determined by quantitative RT-PCR. In this analysis, the target gene expression without stimulation with LPS was set to 1. Click here for file
