# Supplementary material for: Expression analysis of inflammasomes in experimental models of inflammatory and fibrotic liver disease
Source: J Inflamm (Lond). 2012 Nov 28;9:49. doi: 10.1186/1476-9255-9-49 (PMC3599703; doi:10.1186/1476-9255-9-49)
Supplement: Additional file 1 — Statistics to Figure 2. Expression of inflammasome components in rat cirrhotic fat storing cell line CFSC-2G subjected to LPS stimulation. Statistics to Figure 3: Expression of inflammasome components in rat livers after bile duct ligation (BDL). Statistics to Figure 4: Expression of inflammasome components in rat livers after application of CCl4 . Statistics to Figure 5: Expression of inflammasomes in mice after Con A injection. Statistics to Figure 6: Expression of inflammasomes in primary murine hepatocytes after stimulation with LPS. Statistics to Suppl. Figure 3: Induction of acute phase response in mice after LPS injection. [file 1476-9255-9-49-S1.doc]

**Supplementary Material**

**Statistics**

Statistics to Figure 2: Expression of inflammasome components in rat cirrhotic fat storing cell line CFSC-2G subjected to LPS stimulation.

|  | **30 min** | | | **1 h** | | | **2 h** | | |
| --- | --- | --- | --- | --- | --- | --- | --- | --- | --- |
|  | 50 ng | 100 ng | 200 ng | 50 ng | 100 ng | 200 ng | 50 ng | 100 ng | 200 ng |
| **NLRP-1** | 0.4546 | 0.6357 | 0.2891 | 0.6202 | 0.1086 | 0.1322 | 0.0066 | 0.2984 | 0.3040 |
| **NLRP-3** | 0.3336 | 0.6923 | 0.2070 | 0.0437 | 0.0086 | 0.0005 | 0.4735 | 0.1335 | 0.0198 |
| **NLRC4** | 0.3526 | 0.4795 | 0.0710 | 0.0441 | 0.0342 | 0.3701 | 0.2420 | 0.2921 | 0.5842 |
| **AIM-2** | 0.5640 | 0.9696 | 0.2908 | 0.0968 | 0.0001 | 0.0089 | 0.0280 | 0.0351 | 0.0248 |
| **IL-1β** | 0.3728 | 0.0001 | 0.0243 | 0.1263 | 0.1305 | 0.0553 | 0.1279 | 0.1321 | 0.0002 |
| **IL-18** | 0.3671 | 0.3869 | 0.0005 | 0.2749 | 0.4402 | 0.0021 | 0.2609 | 0.1570 | 0.1716 |
| **ASC** | 0.6385 | 0.8407 | 0.0998 | 0.2791 | 0.2326 | 0.6905 | 0.4642 | 0.7419 | 0.0007 |
| **TNF-α** | 0.1369 | 0.1079 | 0.0913 | 0.0073 | 0.0429 | 0.0133 | 0.0506 | 0.0810 | 0.0175 |

|  | **4 h** | | | **8 h** | | | **16 h** | | |
| --- | --- | --- | --- | --- | --- | --- | --- | --- | --- |
|  | 50 ng | 100 ng | 200 ng | 50 ng | 100 ng | 200 ng | 50 ng | 100 ng | 200 ng |
| **NLRP-1** | 0.0031 | 0.0109 | 0.1445 | 0.0518 | 0.1964 | 0.3422 | 0.8820 | 0.3508 | 0.7925 |
| **NLRP-3** | 0.0016 | 0.0008 | 0.2418 | 0.0047 | 0.0796 | 0.0383 | 0.0348 | 0.0092 | 0.0079 |
| **NLRC4** | 0.9435 | 0.2405 | 0.3006 | 0.1162 | 0.0528 | 0.0747 | 0.0074 | 0.0029 | 0.0006 |
| **AIM-2** | 0.3994 | 0.2625 | 0.0023 | 0.1906 | 0.1222 | 0.9265 | 0.5040 | 0.0055 | 0.7924 |
| **IL-1β** | 0.0038 | 0.0347 | 0.0130 | 0.5445 | 0.0700 | 0.4748 | 0.2920 | 0.1330 | 0.0477 |
| **IL-18** | 0.4897 | 0.0908 | 0.2547 | 0.9390 | 0.42008 | 0.9774 | 0.0092 | 0.0006 | 0.0043 |
| **ASC** | 0.0095 | 0.0694 | 0.0091 | 0.4896 | 0.1708 | 0.2653 | 0.4158 | 0.0018 | 0.1186 |
| **TNF-α** | 0.0056 | 0.1360 | 0.0360 | 0.0153 | 0.0038 | 0.0001 | 0.0857 | 0.0068 | 0.0043 |

**Statistics to Figure 3: Expression of inflammasome components in rat livers after bile duct ligation (BDL).**

|  | **2 days** | **7 days** | **14 days** |
| --- | --- | --- | --- |
| **NLRP-1** | 0.0002 | 0.0011 | 0.0025 |
| **NLRP-3** | 0.0002 | 0.0008 | 0.0003 |
| **NLRC4** | 0.0061 | 0.0018 | 0.0006 |
| **AIM-2** | 0.5257 | 0.0820 | 0.2318 |
| **IL-1β** | 0.1754 | 0.1812 | 0.2445 |
| **IL-18** | 0.5176 | 0.2139 | 0.0984 |
| **ASC** | 0.8774 | 0.0492 | 0.0386 |
| **TNF-α** | 0.6969 | 0.0387 | 0.2634 |

**Statistics to Figure 4: Expression of inflammasome components in rat livers after application of CCl_4_**.

|  | **Oil *vs*. CCl_4_ (1x)** |
| --- | --- |
| **NLRP-1** | 0.1365 |
| **NLRP-3** | 0.0844 |
| **NLRC4** | 0.0280 |
| **AIM-2** | 0.0650 |
| **IL-1β** | 0.0033 |
| **IL-18** | 0.2466 |
| **ASC** | 0.0416 |
| **TNF-α** | 0.1932 |
| **IL-1α** | 0.6816 |
| **IL-6** | 0.0246 |
| **IL-10** | 0.0660 |
| **IFN-γ** | 0.2583 |
| **CCL-2** | 0.2457 |

**Statistics to Figure 5: Expression of inflammasomes in mice after Con A injection.**

|  | **Control *vs.* Con A** |
| --- | --- |
| **NLRP-1** | 0.6670 |
| **NLRP-3** | 0.0026 |
| **NLRC4** | 0.0069 |
| **AIM-2** | 0.0606 |
| **IL-1β** | 0.0048 |
| **IL-18** | 0.0004 |
| **ASC** | 0.1861 |
| **TNF-α** | 0.0023 |

Statistics to Figure 6: Expression of inflammasomes in primary murine hepatocytes after stimulation with LPS.

|  | **30 min** | | | | **1 h** | | | |
| --- | --- | --- | --- | --- | --- | --- | --- | --- |
|  | **50** | **100** | **200** | **400** | **50** | **100** | **200** | **400** |
| **NLRP-1** | 0.8 | 0.7 | 0.4 | 0.7 | 0.4 | 0.05 | 0.1 | 0.5 |
| **NLRP-3** | 0.7 | 0.5 | 0.5 | 0.4 | 0.01 | 0.03 | 0.06 | 0.1 |
| **NLRC4** | 0.5 | 0.8 | 0.8 | 0.6 | 0.2 | 0.4 | 0.9 | 0.3 |
| **AIM-2** | 0.5 | 0.6 | 0.5 | 0.5 | 0.3 | 0.2 | 0.4 | 0.3 |
| **IL-1β** | 0.9 | 0.8 | 0.5 | 0.5 | 0.01 | 0.03 | 0.06 | 0.1 |
| **IL-18** | 0.7 | 0.7 | 0.9 | 0.2 | 0.3 | 0.1 | 0.8 | 0.2 |
| **ASC** | 0.7 | 0.6 | 0.8 | 0.9 | 0.7 | 0.6 | 0.5 | 0.09 |
| **TNF-α** | 0.3 | 0.2 | 0.2 | 0.2 | 0.01 | 0.02 | 0.03 | 0.09 |
|  | | | | | | | | |
|  | **2 h** | | | | **4 h** | | | |
|  | **50** | **100** | **200** | **400** | **50** | **100** | **200** | **400** |
| **NLRP-1** | 0.1 | 0.04 | 0.06 | 0.8 | 0.002 | 0.0007 | 0.008 | 0.002 |
| **NLRP-3** | 0.007 | 0.08 | 0.07 | 0.09 | 0.02 | 0.1 | 0.05 | 0.1 |
| **NLRC4** | 0.5 | 0.3 | 0.3 | 0.5 | 0.3 | 0.08 | 0.5 | 0.8 |
| **AIM-2** | 0.2 | 0.3 | 0.1 | 0.3 | 0.2 | 0.4 | 0.5 | 0.8 |
| **IL-1β** | 0.04 | 0.01 | 0.01 | 0.04 | 0.008 | 0.05 | 0.04 | 0.04 |
| **IL-18** | 0.2 | 0.2 | 0.07 | 0.01 | 0.1 | 0.5 | 0.8 | 0.5 |
| **ASC** | 0.8 | 0.5 | 0.7 | 0.3 | 0.01 | 0.02 | 0.009 | 0.0003 |
| **TNF-α** | 0.02 | 0.03 | 0.01 | 0.1 | 0.07 | 0.03 | 0.1 | 0.1 |

Statistics to Suppl. Figure 3: Induction of acute phase response in mice after LPS injection.

|  | **2 h** | **6 h** |
| --- | --- | --- |
| **NLRP-1** | 0.0139 | 0.8285 |
| **NLRP-3** | 0.0207 | 0.0546 |
| **NLRC4** | 0.1330 | 0.2392 |
| **AIM-2** | 0.2274 | 0.0596 |
| **IL-1β** | 0.0070 | 0.0543 |
| **IL-18** | 0.3119 | 0.0010 |
| **ASC** | 0.9769 | 0.0509 |
| **TNF-α** | 0.0190 | 0.0412 |
| **IL-1α** | 0.0319 | 0.1193 |
| **IL-6** | 0.0256 | 0.0689 |
| **IL-10** | 0.0220 | 0.0700 |
| **IFN-γ** | 0.0729 | 0.0752 |
| **CCL-2** | 0.0044 | 0.0670 |
