# Supplementary figures and images for: Expression analysis of inflammasomes in experimental models of inflammatory and fibrotic liver disease
Source: J Inflamm (Lond). 2012 Nov 28;9:49. doi: 10.1186/1476-9255-9-49 (PMC3599703; doi:10.1186/1476-9255-9-49)

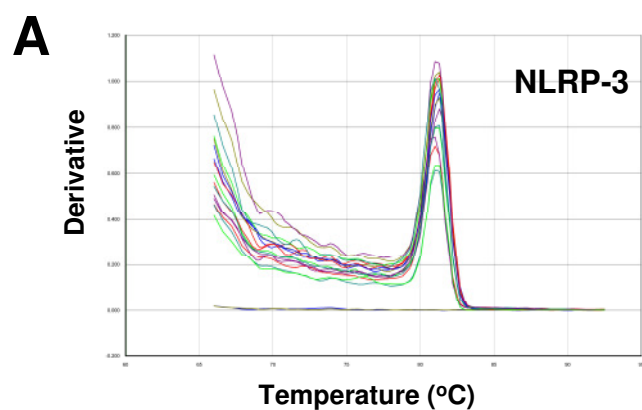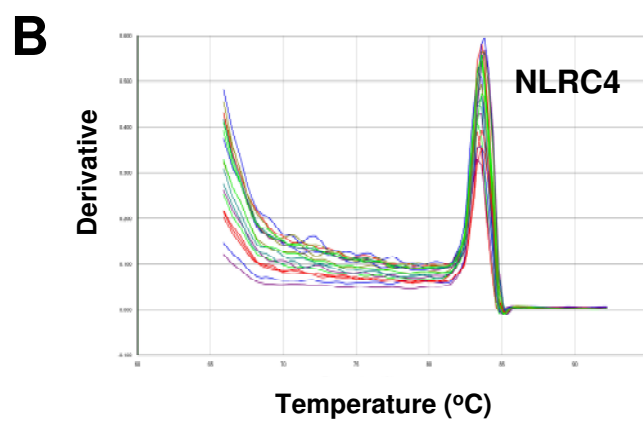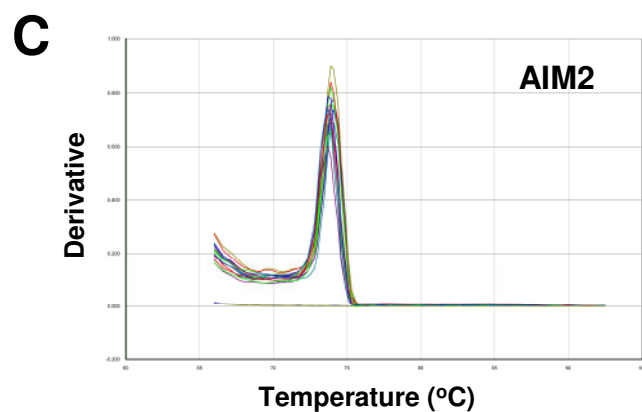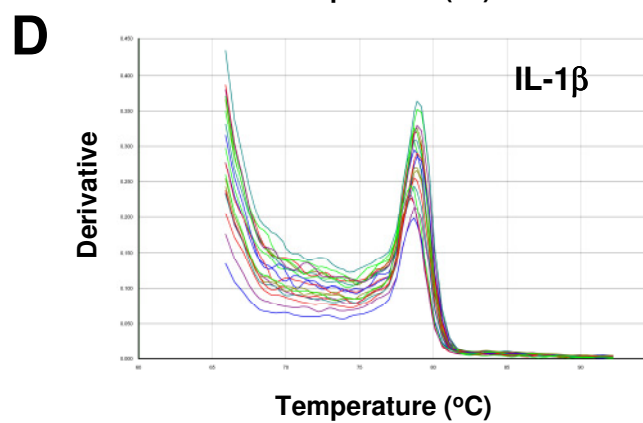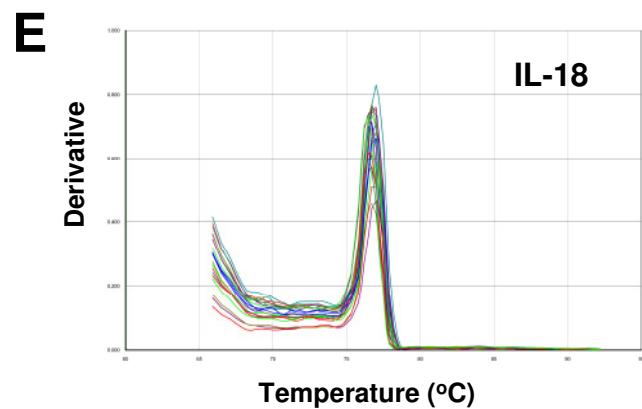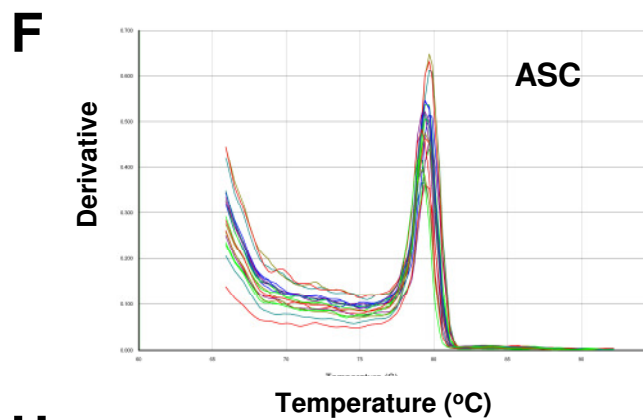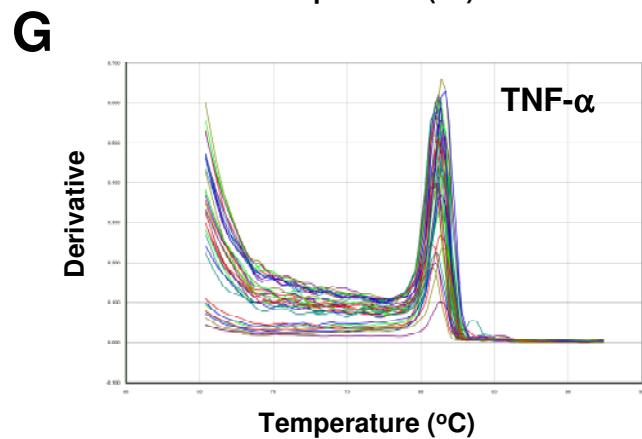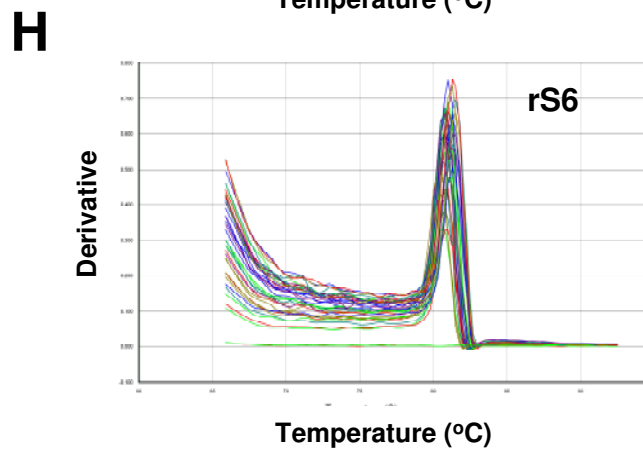

Supplement: Additional file 2: Figure S1 — Establishment of TaqMan tests for expression analysis of individual inflammasome genes in rats. TaqMan assays for rat NLRP-3 (A), NLRC-4 (B), AIM2 (C), IL-1β (D), IL-18 (E), ASC (F), TNF-α (G), and rS6 (H) were established. Representative melting curves for each gene are depicted. Amplification of respective target gene sequences were performed under the same cycling conditions using a melting temperature of 95°C and amplification/extension temperatures of 60°C, respectively. The individual primer combinations used in each test are depicted in Table 1. [file 1476-9255-9-49-S2.pdf]

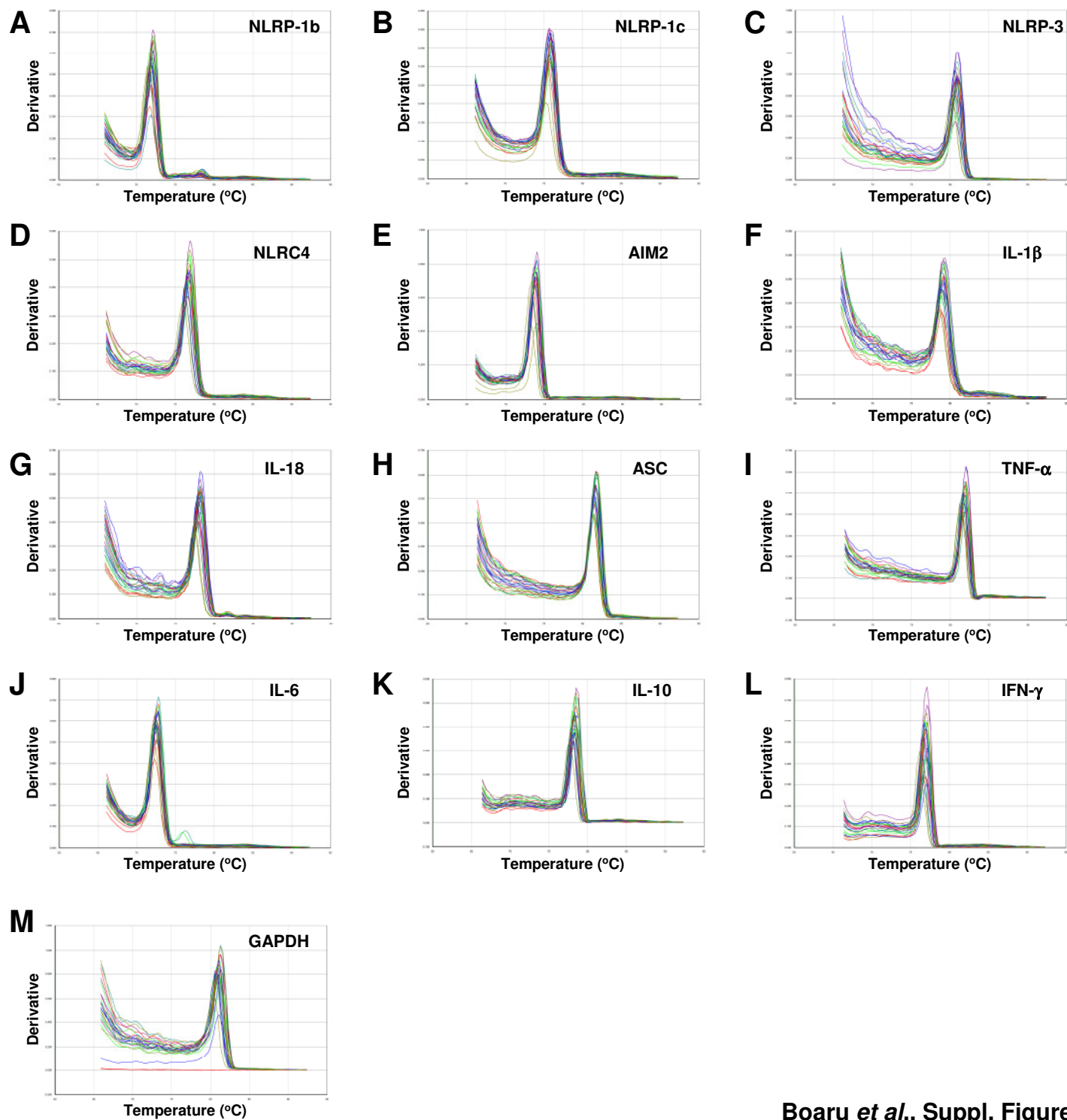

Boaru *et al.*, Suppl. Figure 2

Supplement: Additional file 3: Figure S2 — Establishment of TaqMan tests for expression analysis of individual inflammasome genes in mouse. TaqMan assays for murine NLRP-1b (A), NLRP-1c (B), NLRP-3 (C), NLRC-4 (D), AIM2 (E), IL-1β (F), IL-18 (G), ASC (H), TNF-α (I), IL-6 (J), IL-10 (K), IFN-γ (L) and GAPDH (M) were established. Amplification of the different gene sequences were essentially performed under the same cycling conditions each using a melting temperature of 95°C and amplification/extension temperatures of 60°C, respectively. Representative melting curves for each gene are depicted. The individual primer combinations used in each test are depicted in Table 1. [file 1476-9255-9-49-S3.pdf]
